# Supplementary material for: Active colloids as mobile microelectrodes for unified label-free selective cargo transport
Source: Nat Commun. 2018 Feb 22;9:760. doi: 10.1038/s41467-018-03086-2 (PMC5823901; doi:10.1038/s41467-018-03086-2)
Supplement: Supplementary file 1 — Supplementary Information [file 41467_2018_3086_MOESM1_ESM.docx]

Supplementary Figure 1: Schematic showing geometry of Janus particle and its first image (dotted line).


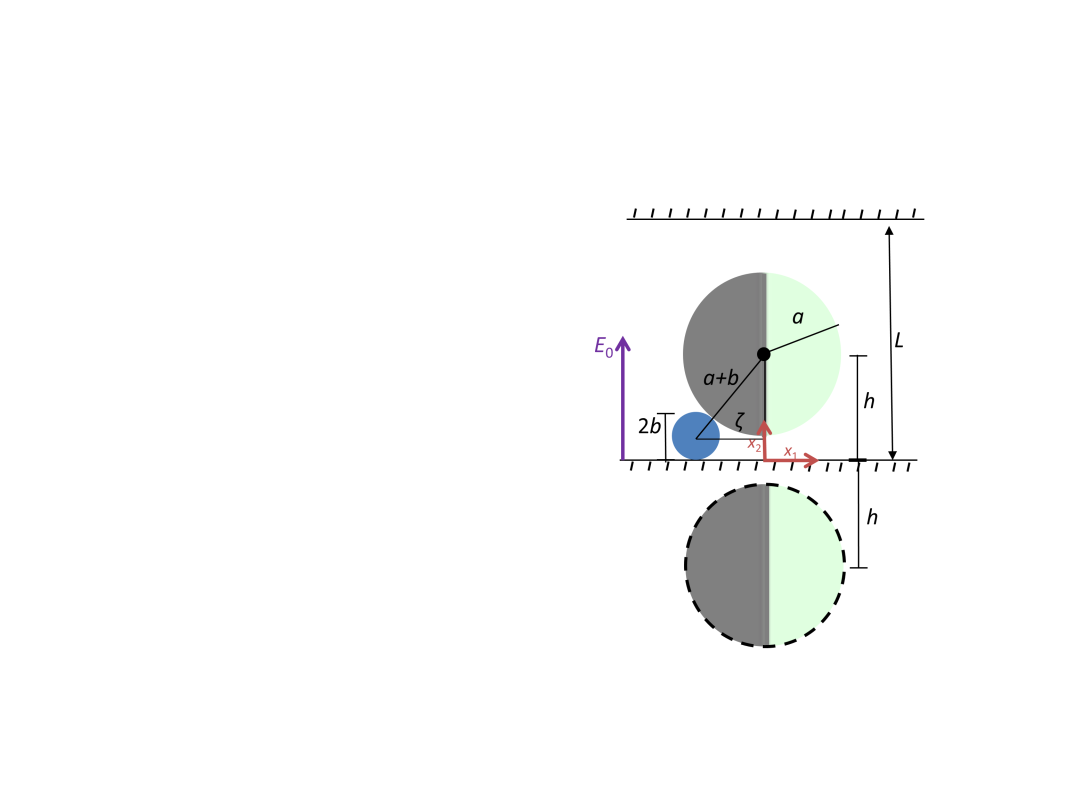

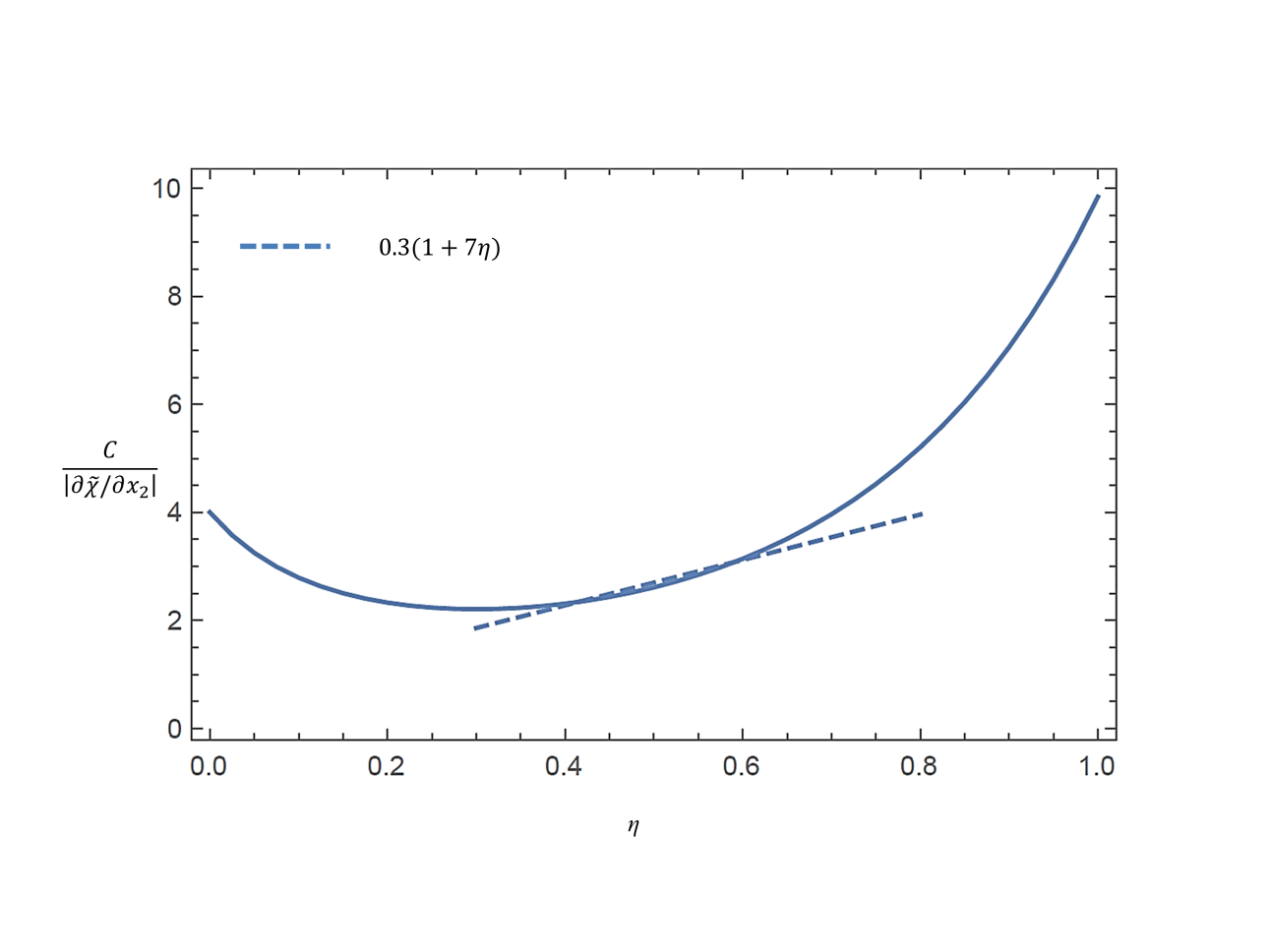


Supplementary Figure 2: Linear approximation for the inverse of the induced electric field

**Supplementary Note 1: Scaling analysis of the electric field and DEP trapping**

The accumulation of a target at the surface of the Janus particle is dictated by the magnitude and spatial distribution of the induced electric field, as the dielectrophoretic trapping mechanism is proportional to the electric field gradient. Essentially, the trapping of the target, represents the dominance of the dielectrophoretic potential, evaluated at the center of the target (in this case, located at $\left(x_{1},b,0\right)$ (Supplementary Figure 1)) over the thermal potential (Brownian motion)^1^ such that,

,

where $k_{\text{B}}$ is the Boltzmann constant, $T$ the temperature and $\Delta$ represents the ratio of thermal and dielectrophoretic potentials (generally presumed to be at least an order of magnitude, i.e., $\Delta\sim10$^1^) , left here as a single fitting parameter for the experimental data.

Following Jones^2^ to first order, the dielectrophoretic force on the target can be written in terms of the externally applied electric field, $\mathbf{E}$as

$\mathbf{F}_{\text{DEP}}=2\pi b^{3}\varepsilon_{\text{f}}\mathrm{Re}\left\{K_{\text{CM}}\right\}\left(\mathbf{E}\cdot\nabla\right)\mathbf{E}$,

in which $b$ is the radius of the target, $\varepsilon_{\text{f}}$is the permittivity of the fluid and $K_{\text{CM}}$ is the Clausius-Mossotti factor of the target, prescribed by

$$K_{\text{CM}}=\frac{\tilde{\varepsilon}_{\text{p}}-\tilde{\varepsilon}_{\text{f}}}{\tilde{\varepsilon}_{\text{p}}+2\tilde{\varepsilon}_{\text{f}}},$$

where $\tilde{\varepsilon}_{\text{p,f}}$ are the complex permittivities of the particle and fluid respectively, defined in terms of the real permittivity and conductivity according to$\tilde{\varepsilon}_{\text{p,f}}=\varepsilon_{\text{p,f}}+\sigma_{\text{p,f}}/i\omega$. Experimentally, it has been determined that although the Clausius-Mossotti factor only accounts for material properties, in some cases, such as dielectric nanoparticles, the CM is actually dependent on geometry and surface charge. Most commonly this is accounted for by correcting the material conductivity $\sigma_{p}$ to an effective conductivity $\sigma'_{\text{p}}$ accounting for surface conductance such that $\sigma'_{\text{p}}=\sigma_{\text{p}}+K_{\text{s}}/a$ where $K_{s}$ is the surface charge and $a$ is the particle radius^3,4^.

Generally, for a uniform applied field$\left(\mathbf{E}\cdot\nabla\right)\mathbf{E}=0$ and one would not expect the target to experience a dielectrophoretic force. In the current case however, the polarization of the Janus particle results in a locally non-uniform field such that the solution for the potential in the bulk fluid must satisfy^5^

,

where $Q$ is the induced charge distribution, $\Omega_{\text{MW}}=\omega\lambda_{0}^{2}/D$ (where $\omega[\text{rads}^{\text{-1}}]$ is the frequency of the applied field, $\lambda_{0}[\text{m}]$ is the thickness of the EDL, $D[\text{m}^{2}\mathrm{s}^{-1}]$ is the diffusivity of the symmetric electrolyte) and $\chi$ is an harmonic function. In the limit of a thin EDL, i.e., $\lambda_{0}/a\to0$, $Q\to0$ and .

For the current configuration, of a Janus sphere, whose center is located at a distance $\left(0,h,0\right)$ from a conducting wall we prescribe the disturbance potentials induced both by the Janus particle itself, $\chi_{\text{JP}}$ and that of the first image, $\chi'$ located at $\left(0,-h,0\right)$^6^. Here we have designated the origin of the Cartesian coordinate system $\left(x_{1},x_{2},x_{3}\right)$ to be on the wall, directly below the interface of the JP (Supplementary Figure 1).

Following^6,7^, the total induced potential, $\chi$, around a single JP and its image, prescribed by $\chi_{\text{JP}}$ and $\chi'$, respectively, subject to a uniform applied field of unit amplitude in the $x_{2}$ direction is given by

$\chi=-2x_{2}+\tilde{\chi}, \tilde{\chi}=\chi_{\text{JP}}+\chi'$,

where

$\begin{matrix} \chi_{\text{JP}}=-\frac{P_{1}a^{3}\left(x_{2}-h\right)}{R^{3}}+\frac{6P_{2}a^{4}x_{1}\left(x_{2}-h\right)}{R^{5}} ;R^{2}=x_{1}^{2}+\left(x_{2}-h\right)^{2} \\ \chi'=-\frac{P_{1}a^{3}\left(x_{2}+h\right)}{R'^{3}}+\frac{6P_{2}a^{4}x_{1}\left(x_{2}+h\right)}{R'^{5}} ;\left(R'\right)^{2}=x_{1}^{2}+\left(x_{2}+h\right)^{2} \end{matrix}$,

in which$P_{1},P_{2}$ can be considered the strength of a point dipole and quadrupole, respectively, whose frequency dependent magnitudes are obtained from the applied boundary conditions used to solve the electrostatic problem. In the limit of a homogeneous infinitely polarizable particle, $P_{1}=1$ and $P_{2}=0$ while at high frequency$\left(\omega\lambda_{0}a/D>1\right)$, a Janus sphere with large dielectric contrast between the two hemispheres will be characterized by $P_{1}\sim-1,P_{2}\sim-0.4$^7,8^. Within this definition, the total potential satisfies the equipotential boundary condition at the conducting wall$\left(x_{2}=0\right)$, including in the far-field condition where$\chi\to-2x_{2}$.

In order to obtain the dielectrophoretic force exerted by the non-uniformity induced by the presence of the Janus particle, we simply note following Eq.(2) and Eq.(5) that $\left(\mathbf{E}\cdot\nabla\right)\mathbf{E}=\frac{1}{2}\nabla\left|\mathbf{E}\right|^{2}=\frac{1}{2}E_{0}^{2}\nabla\left|\nabla(-x_{2}+\tilde{\chi}/2)\right|^{2}\sim\frac{1}{2}E_{0}^{2}\nabla\left|\frac{\partial\tilde{\chi}}{\partial x_{2}}\right|$. Accordingly, we can rewrite the DEP force defined in in terms of the leading contribution of the electric potential $U$induced by the presence of the Janus sphere

$F_{\text{DEP}}\sim\nabla U_{\text{DEP}};\text{ }U_{\text{DEP}}\sim2\pi b^{3}\varepsilon_{\text{f}}\mathrm{E}_{0}^{2}\mathrm{Re}\left\{K_{\text{CM}}\right\}\left|\frac{1}{2}\frac{\partial\tilde{\chi}}{\partial x_{2}}\right|+const.$.

Substituting into yields the scaling of the voltage as a function of the induced electric field as

$V_{\text{RMS}}^{2}=\left(E_{0}L\right)^{2}\sim\frac{C}{\left|\partial\tilde{\chi}/\partial x_{2}\right|};C=\frac{\Delta k_{\text{B}}TL^{2}}{\pi\varepsilon_{\text{f}}b^{3}\mathrm{Re}\left\{K_{\text{CM}}\right\}}$.

For $L=120\text{μm}$, $k_{\text{B}}=1.38\times10^{-23}\text{m}^{2}\text{kgs}^{\text{-2}}\text{K}^{\text{-1}}$, $T=298\text{K}$, $\varepsilon_{\text{f}}=80\cdot8.85\times10^{-12}\text{Fm}^{\text{-1}}$, $\mathrm{Re}\left\{K_{\text{CM}}\right\}\sim1$ (high frequency) and $b=150\text{nm}$, $C=8\Delta\text{V}_{RMS}^{2}$.

In order to extract a simplified scaling for the induced electric field, acting on a target centered at $\left(x_{1},b,0\right)$ (Supplementary Figure 1), we make a number of approximations for $\partial\tilde{\chi}/\partial x_{2}$. Specifically, for a small gap between the Janus particle and the substrate (generally on the order of at most a few 100nm), $h\sim a$, while the small size of the target implies $b/a<<1$ such that we can approximate $\left|x_{2}\pm h\right|\sim a$. Noting that trapping occurs at the side of the metallic hemisphere ($x_{1}<0$) and that in most cases, it does not tend to extend further than the radius of the JP, i.e., $\frac{x_{1}}{a}<1$, it is convenient therefore to express $\frac{\partial\tilde{\chi}}{\partial x_{2}}$ in terms of a nondimensional parameter $\eta=\left|\frac{x_{1}}{a}\right|$ as

$$\frac{\partial\tilde{\chi}}{\partial x_{2}}\sim2\left(\frac{P_{1}\left(2-\eta^{2}\right)}{\left(1+\eta^{2}\right)^{5/2}}+\frac{6P_{2}\eta\left(4-\eta^{2}\right)}{\left(1+\eta^{2}\right)^{7/2}}\right)+\left(h.o.m\right)$$

where (h.o.m) denotes higher order multipoles. Noting that the area, $A$, measured in part d of Figure 2 is approximately hemispherical, we can extract the dependence of the maximum value of $\eta$ at which the target is trapped on the voltage by defining $\eta_{\max}^{2}=\frac{x_{1,\max}^{2}}{a^{2}}\sim\frac{2A+\pi\zeta^{2}}{\pi a^{2}}$. Here, $\zeta$ represents the minimum value of $\eta$, dictated by the geometry of the problem and corresponds to the minimum distance from the center of the JP which the target can reach (Supplementary Figure 1)

$\zeta=-\sqrt{\left(a+b\right)^{2}-\left(h-b\right)^{2}}$.

For a small gap ($h\sim a$), $\zeta=-2\sqrt{ba}$ which corresponds to $\left.\eta\right|_{\zeta}=\eta_{\min}=2\left(b/a\right)^{1/2}$. In this regime, where for the current experimental parameters $0.3<\eta<0.6$, Eq. can be further simplified by using a linear approximation (plotted in Supplementary Figure 2) for such that

$$C/\left|\partial\tilde{\chi}/\partial x_{2}\right|\sim0.3\left(1+7\eta\right)$$

Using a least squares regression, we get a linear fit at low voltages with gradient corresponding to values of $\Delta\sim8$. In quantitative terms, the value of $\Delta$ is generally presumed to be at least ten, such that the dielectrophoretic potential is approximately an order of magnitude greater than the thermal potential^1^. At high voltages, we observe that the trapping plateaus at lower values of $\eta$ than theoretically predicted by Eq.. This could be due to the fact that $h/a>1$ as well as particle-particle interactions and 3D shielding.

**Supplementary References**

1. Hughes, M. P. & Morgan, H. Dielectrophoretic trapping of single sub-micrometre scale bioparticles. *J. Phys. Appl. Phys.* **31,** 2205 (1998).

2. Jones, T. B., *Electromechanics of Particles*. (Cambridge University Press, 2005).

3. Honegger, T., Berton, K., Picard, E. & Peyrade, D. Determination of Clausius–Mossotti factors and surface capacitances for colloidal particles. *Appl. Phys. Lett.* **98,** 181906 (2011).

4. Green, N. G. & Morgan, H. Dielectrophoresis of Submicrometer Latex Spheres. 1. Experimental Results. *J. Phys. Chem. B* **103,** 41–50 (1999).

5. Miloh, T. Nonlinear alternating electric field dipolophoresis of spherical nanoparticles. *Phys. Fluids 1994-Present* **21,** 072002 (2009).

6. Miloh, T. Dipolophoresis of Janus nanoparticles in a microchannel. *Electrophoresis* **34,** 1939–1949 (2013).

7. Boymelgreen, A. M. & Miloh, T. Induced-charge electrophoresis of uncharged dielectric spherical Janus particles. *Electrophoresis* **33,** 870–879 (2012).

8. Boymelgreen, A., Yossifon, G. & Miloh, T. Propulsion of Active Colloids by Self-Induced Field Gradients. *Langmuir* **32,** 9540–9547 (2016).
