# Supplementary material for: Active colloids as mobile microelectrodes for unified label-free selective cargo transport
Source: Nat Commun. 2018 Feb 22;9:760. doi: 10.1038/s41467-018-03086-2 (PMC5823901; doi:10.1038/s41467-018-03086-2)
Supplement: Supplementary file 3 — Description of Additional Supplementary Files [file 41467_2018_3086_MOESM3_ESM.pdf]

Description of Additional Supplementary Files

File Name: Supplementary Movie 1
Description: Accumulation, transport and release of target.
Video corresponds to Figure 3a and shows a 15um Janus particle transporting 300nm Ps target at 100kHz and releasing them at 2MHz.

File Name: Supplementary Movie 2
Description: Selective assembly and transport of target.
Video corresponds to Figure 3d and shows a 15um Janus particle transporting 300nm and 1um Ps targets at 1kHz and at 100kHz, releasing the 1um at 750kHz and travelling with only the 300nm and finally realizing the 300nm at 2MHz.
